# The Most and Least Stressful Prehospital Emergencies from Emergency Medical Technicians’ View Point; a Cross-Sectional Study

**Published:** 2019-02-15

**Authors:** Afshin Khazaei, Maryam Esmaeili, Elham Navab

**Affiliations:** 1 Intensive Care and Management Nursing Department, School of Nursing and Midwifery, Tehran University of Medical Sciences, Tehran, Iran.; 2 Critical Care and Geriatric Nursing Department, School of Nursing and Midwifery, Tehran University of Medical Sciences, Tehran, Iran.

**Keywords:** Emergency medical services; emergency medical technicians, stress disorders, post-traumatic, mental disorders, emergency treatment

## Abstract

**Introduction:**

Exposure to different prehospital emergencies (PE) may have a different impact on Emergency Medical Technicians (EMTs) based on the characteristics and circumstances of the emergency. The present study aimed to prioritize PE types according to their stressfulness as well as their correlation with post traumatic stress disorders (PTSD).

**Method:**

In this cross-sectional study, all EMTs in the Emergency Medical Services (EMS) of Hamadan province were invited to participate, voluntarily. The study questionnaire comprised of two parts: a) personal characteristics and prioritizing PE types in terms of their stressfulness and b) The PTSD checklist. Kruskal-Wallis test was used for examining the relationship between total PTSD score and the most and the least stressful PEs. Multivariate logistic regression was also used to predict the impact of different types of PEs on PTSD.

**Results:**

259 EMTs with the mean age of 32.79±6.16 years were studied. The total mean score of PTSD on PCL-5 was 21.60±11.45. Also, 20.1% of technicians met the criteria for PTSD. The mean age of technicians who met PTSD criteria was less than that of technicians who did not meet PTSD criteria (28 vs. 33 years, P<0.001). The most and least stressful emergencies were cardiovascular (24.7%) and environmental (26.3%) emergencies, respectively. There was a significant correlation between the most (Kruskal-Wallis=40.92, df=12, p < 0.001) and the least stressful emergencies (Kruskal-Wallis=28.22, df=15, p = 0.02) from EMTs’ viewpoint and PTSD score. Multivariate logistic analysis showed that gynecologic (aOR=2.28, Wald=5.83, p=0.016) and allergic (aOR=0.12, Wald=10.16, p=0.01) emergencies were significant predictive factors of PTSD.

**Conclusion:**

Based on the view point of the studied EMTs, cardiac and environmental emergencies were the most and least stressful emergencies. The frequency of PTSD in this series was about 20% and it significantly correlated with younger age, lower experience, higher number of shifts, non-official employment and EMT degree. Based on multivariate logistic analysis, gynecologic and allergic emergencies were the only significant predictive factors of PTSD.

## Introduction:

Emergency Medical Technicians (EMTs) experience significant stress at work (1). The cumulative stresses experienced can be associated with traumatic events and cause traumatic stresses (2). Traumatic or critical events often refer to incidents in which EMTs experience severe and acute stress in the face of situations like witnessing the death of patients (including children), feeling unable to help the patient and feeling at risk (3). Frequent and repeated exposure to potentially traumatic events may expose EMTs to serious psychiatric compromise such as post-traumatic stress disorder (PTSD) (4, 5). PTSD is a complex physical, cognitive, emotional, behavioural and psychological disorder, which is associated with intrusive thoughts, nightmares, avoiding reminders of a trauma, anxiety, and sleep disturbance, which ultimately leads to social, occupational and interpersonal disorders (6). While efforts have been made to identify the critical or traumatic events in the prehospital setting, so far a comprehensive review of various types of the prehospital emergencies (PEs) in term of their stressful situations has not been done and studies in this area are still sparse. Donnelly et al., for example, identified 29 incidents that were considered traumatic events by technicians, such as seeing a death scene, seeing the scene where patients have been beaten, threatened with weapons, the scene where technicians have been threatened, exposure to toxic substances, and severe vehicle accidents (7). Therefore, exposure to different PEs may have a different impact on technicians based on the characteristics and circumstances of the emergency (unpredictable, non-controllable, and dynamic environment). For example, Guise et al. showed in their study that pediatric emergencies would cause the most stress and anxiety in EMTs (8). Bostrom et al. also introduced obstetric and gynecologic emergencies under uncertain prehospital conditions as the most stressful mission for EMTs (9). In Iran, emergency missions for which EMTs are dispatched include cardiovascular, environmental, respiratory, shock, obstetric and gynecologic, neurological, toxicity, metabolic, burning, accidents, acute abdominal and bleeding emergencies (10, 11). One of the questions that are not yet comprehensively answered is that exposure to which type of pre-hospital emergency causes the most and least stress from the technicians' perspective.

 With the current overall PTSD prevalence (from 11% to 35%) among EMTs (12, 13), which is the highest among rescue workers (including police or firefighters) (14), assessing the mental health of EMS providers and identifying staff at high risk of developing PTSD is vital. Also, identifying the order in which PE types associate with PTSD can be effective in development of practical approaches to reduce the stress among EMS staff. Therefore, the present study aimed to prioritize PE types according to their stressfulness as well as their correlation with post traumatic stress disordes (PTSD).

## Method:

***Study design and setting***

This study is a multicenter, cross-sectional study, conducted between July 2018 and October 2018. The study population comprised of EMTs in emergency bases in Hamadan province (in west of Iran) that has 20 metropolitan bases, 30 road bases, and one air base, which serve a population of about two million. All EMTs in the Emergency Medical Services (EMS) of Hamadan province were invited to take part in our study, voluntarily. Information about the study was orally given to the EMTs before its initiation. Also, the participants were assured that their names and personal information would be kept confidential. The research project was approved by the Ethical Committee of the School of Nursing and Midwifery and School of Rehabilitation of Tehran University of Medical Sciences (No: IR.UMSHA.REC.1396.808). 

***Participant***

The total number of EMTs in Hamadan at the time of data collection was 307. The active EMTs who were present in urban, road and air emergency bases on a full-time basis and announced their oral and written approvals were included in the study. Non-active EMS personnel, personnel from other medical centers working part time in the EMS and the personnel who had experienced non-occupational stressors, such as the death of close relatives, etc. in the previous eight weeks were excluded from the study.

***Data gathering***

The study questionnaire comprised of two parts: a) personal characteristics and prioritizing pre-hospital emergency types in terms of their stressfulness and b) The PTSD checklist, which was a 20-item self-report measure that assessed the presence and severity of PTSD symptoms. Items on the PTSD Checklist for DSM-5 (PCL-5) correspond with DSM-5 criteria for PTSD, which is intended to assess the individual’s symptoms in the past month (15). 

The PCL-5 checklist has 20-items divided into four clusters including; B- Intrusion (five items), C- avoidance (two items), D- negative alterations in cognition and mood (seven items) and E- alterations in arousal and reactivity (six items). Also, the items of the PTSD checklist are rated on a 5-point Likert-type scale (0=“not at all” to 4=“extremely”). Therefore, a total PTSD score can be obtained by summing up the scores of the 20 items (ranges from 0 to 80). Also, PTSD diagnosis via a PCL-5 cut-point score, which is the score of 33, seems logical when further psychometric testing is not available (15-17). Therefore, we divided PTSD scores into score≥33 (met the criteria for PTSD) and score<33 (did not meet the criteria for PTSD) for screening.

The questionnaires were given to the participants by the researcher and collected after completion. Technicians were asked to report the most and the least stressful prehospital emergencies from their point of view. Reliability and validity of this checklist have been evaluated in several studies and most of these studies showed the high validity and reliability of this tool in detecting PTSD symptoms (18-20). Validity and reliability of the Persian version of this tool were also tested through factor analysis and Cronbach's alpha (0.79%), as well as retest (0.77%), which were ultimately satisfactory (21). However, we reassessed the reliability of PCL-5 through Cronbach’s α (0.89).

***Statistical analysis***

In a study conducted by Sedigheh and et al. (22), PTSD rate reported in the EMTs was 0.22%. Using this data and taking into account the relative errors of 5% and 95% confidence interval (CI), as well as applying the coefficient of the limited population (a total of 307 active EMTs); a minimum of 251 participants was required. Means and standard deviations were used for reporting normally distributed continuous data. non-normally distributed variables were expressed as median (IQR). Categorical variables were reported as frequency and percentages. Kruskal-Wallis test was used for examining the correlation between total PTSD score and the most and the least stressful prehospital emergencies. The multivariate logistic regression model was also used to predict the impact of different types of prehospital emergencies on PTSD. The forward selection (wald) method was used to select covariates for the adjusted models. A receiver operating characteristic (ROC) curve was also drawn for the final adjusted model. All statistical analyses were performed using IBM SPSS Statistics (V. 20). P< 0.05 was considered significant (two-tailed).

## Result:

***Baseline characteristics of studied EMTs***

259 EMTs entered the study after being screened for the inclusion criteria. The mean age of participants was 32.79±6.16 years and their median work experience was 9 years (IQR: 5-12). The mean PTSD score on PCL-5 was 21.60±11.45. Also, 20.1% of the technician met the criteria for PTSD. The mean age of technicians who met PTSD criteria was less than that of technicians who did not meet PTSD criteria (28 vs. 33 years, P<0.001). More details of demographic characteristics in terms of meeting PTSD criteria or not are shown in Table 1.

**Figure 1:**
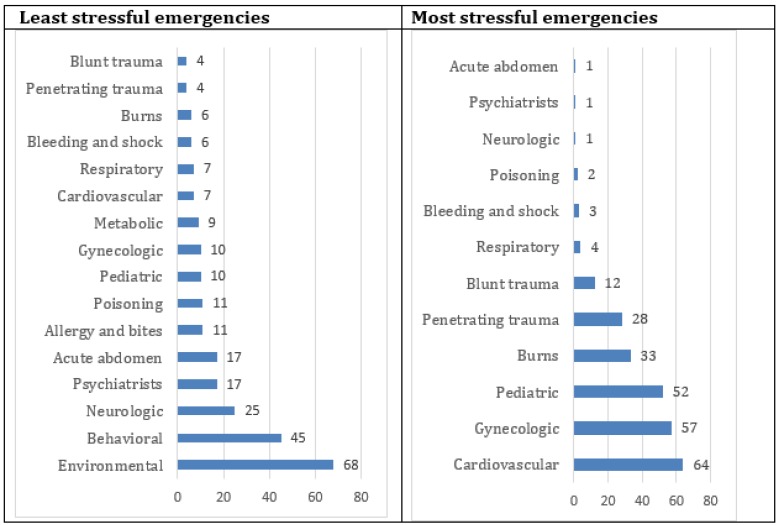
The frequency of participants’ votes to least and most stressful prehospital emergencies

Cardiovascular (24.7%), gynecological (22.0%) and pediatric (20.1%) emergencies, respectively, were most frequently introduced as the most stressful emergencies by the participants. Also, the environmental (26.3%), behavioural (17.4%) and neurological (9.7%) emergencies were most frequently introduced as least stressful emergencies, respectively (figure 1). Also, the technicians based in urban, road and air bases stated that the most stressful emergencies were cardiovascular (21.62%), gynecological (10.42%) and penetrating trauma (1.16%), respectively. 

***PTSD and stressfulness of emergencies***

There was a significant correlation between the most (Kruskal-Wallis=40.92, df=12, p<0.001) and the least stressful emergencies from the viewpoint of EMTs (Kruskal-Wallis=28.22, df=15, p = 0.02) and PTSD score. EMTs who were stationed at the urban bases were more at risk for PTSD. 

**Table 1:** The relationship between demographic characteristics and meeting or not meeting PTSD criteria

**Variable**	**PTSD**		**P**
	**NO (** **score ≥ 33** **)**	**YES (** **score < 33** **)**	
**Age (year)**	33.77 ± 5.56	28.88 (6.94)	< 0.001
**Number of missions (IQR)**	60 (6 - 85)	42 (12 - 110)	0.029
**Work experience (year)**	9.71 ± 4.73	5.12 ± 4.31	< 0.001
**Number of shifts (IQR)**	11 (10-12)	14 (13-15)	< 0.001
**PTSD (Total score) **	17.47±8.36	38.02±6.08	< 0.001
**Base location**			
Urban	136 (82.4)	29 (17.6)	0.079
Road	49 (71.0)	20 (29.0)	
Air	22 (88.0)	3 (12.0)	
**Marital status**			
Married	137 (80.1)	34 (19.9)	0.529
Single	67 (80.7)	16 (19.3)	
Divorced	3 (60.0)	2 (40.0)	
**Employment status**			
Official	85 (87.6)	12 (12.4)	0.017
Non-Official	122 (75.3)	40 (24.7)	
**Degree**			
EMT	96 (73.8)	34 (26.2)	0.038
Operating room technician	27 (77.1)	8 (22.9)	
Anesthesia technician	29 (87.9)	4 (12.1)	
Nurse	55 (90.2)	6 (9.8)	
**In-service training **			
Yes	112 (80.6)	27 (19.4)	0.778
No	95 (79.2)	25 (20.8)	

Data are presented as mean ± standard deviation or number (%). IQR: Interquartile Range.

**Table 2:** The mean of PTSD cluster scores in more stressful and least stressful prehospital emergencies

**Emergencies**	**Intrusion **	**Avoidance **	**Alterations in**	
			**cognitions**	**Arousal & Reactivity **
**More stressful**				
Cardiovascular	5.67±3.22	2.61±1.85	8.44±5.12	7.75±4.14
Gynecological	5.58±3.23	2.51±1.61	8.47±4.72	7.79±4.19
Pediatric	5.31±3.07	2.33±1.59	8.02±4.14	7.62±3.89
**Least stressful**				
Environmental	4.32±2.33	1.97±1.45	6.71±3.88	5.97±3.44
Behavioural	4.98±3.31	2.31±1.88	6.98±4.60	7.20±4.84
Neurological	5.96±3.18	2.44±1.75	9.16±5.39	8.08±4.40

Data are presented as mean ± standard deviation.

Mean work experience in the PTSD and non-PTSD groups were 5.12±4.31 and 9.71±4.73 (P<0.001) (table 1). In terms of PTSD cluster symptoms, in the most and the least stressful PEs, negative alterations in cognitions and intrusion were the higher and lower frequency of PTSD cluster symptoms, respectively (table 2).

After controlling confounding factors, the multivariate logistic analysis showed that gynecologic (aOR=2.28, Wald=5.83, p=0.016) and allergic (aOR=0.12, Wald=10.16, p=0.01) emergencies were significant predictive factors of PTSD. 

## Discussion:

Based on the view point of the studied EMTs, cardiac and environmental emergencies were the most and least stressful emergencies. The frequency of PTSD in this series was around 20% and it significantly correlated with younger age, lower experience, higher number of shifts, non-official employment and EMT degree. Based on multivariate logistic analysis, gynecologic and allergic emergencies were the only factors that significantly correlated with PTSD.

The level of PTSD among EMTs in our study (nearly 20%) was higher than other studies in this field. The results of a systematic review showed that PTSD prevalence rates among ambulance staff was about 11% (23). 

The results of our study indicated that younger people are more vulnerable when exposed to pre-hospital emergencies in comparison with older staff, which was inconsistent with other studies (24). 

Our study showed that cardiac, gynecological and pediatric emergencies were the most stressful emergencies from the viewpoint of EMTs. In contrast, environmental, behavioural and neurological emergencies were the least stressful missions. In this regard, in a qualitative study, Bohstrom et al. showed that going on missions that involved sick children and childbirth were considered as major stress factors by EMTs (9). In their study, Bracken-Scally et al. also stated that from the perspective of most retired EMTs, incidents involving children were particularly difficult to manage (25). Also, in a qualitative study, Froutan et al. showed that pre-hospital burn mission was a unique experience from the perspective of Iranian emergency care staff (26). Cardiac emergency is among the most challenging and frequent missions in the prehospital setting (27), which was selected as the most stressful mission by EMTs in our study. We do not know whether exposure to cardiovascular emergency affects the onset or progression of PTSD in the EMTs or not, but it has been proven that experiencing stressful events induces psychological and physiological stress reactions, which lead to mental disorders (28). Additionally, Compton et al. showed that witnessing unsuccessful out-of-hospital cardiopulmonary resuscitation (CPR) may be associated with displaying symptoms of PTSD in the layperson (24). 

Work experience was another factor in our study that affected PTSD severity. In our study, mean work experience in PTSD group was lower than non-PTSD group. This result was in contradiction with the study of Bezabh et al. that showed the odds of developing PTSD were more than two times higher among EMTs with 4–5 years of work experience compared to those with 3 years of work experience (aOR=2.67;P< 0.001)(29). 

One of the reasons that gynecological emergency is identified as a stressful mission in our study could be that in Iran, EMTs do not participate in any practical training courses on real patients during their study in university and in-service periods and they receive this skill only in theoretical education or on a mannequin. It should be noted that in Iran, only female health care providers can participate in gynecological practice while all the EMTs are male. 

Also, technicians of road bases selected gynecological emergency as a stressful mission. This could be due to the long distances between road bases and medical centers and the probability of mortality and morbidity of the mothers and infants as a result of this delay, the stress of facing this type of emergency is more in the technicians based on road bases. However, carrying out a qualitative study in the pre-hospital emergency is necessary for identifying the various aspects of this issue. EMS managers need to assess PEs for anticipating the possible traumatic events that may affect the EMTs in order to develop appropriate strategies to deal with the negative mental effects on the staff at risk. Therefore, there is a need to carry out more studies, especially a qualitative study, to uncover and provide a thorough description of potential stress in exposure to a variety of PEs.

***Limitations***

Because of the cumulative effect of stresses in exposure to pre-hospital emergencies and heterogeneity of traumatic events, the PTSD in the EMTs cannot be attributed to a special prehospital emergency. In other words, due to the cumulative effect of stress, it is not possible to determine the contribution of each of these emergencies to onset or development of PTSD in EMTs. The results of this study were obtained in our context using multivariate logistic regression after controlling for the possibility of other potentially influential PE and interaction between them. Therefore, the results of this study may not be generalizable to other contexts.

## Conclusions:

Based on the view point of the studied EMTs, cardiac and environmental emergencies were the most and least stressful emergencies. The frequency of PTSD in this series was stimated to be about 20% and this ratio was significantly higher in younger EMTs, those with lower experience, higher number of shifts, non-official employment and EMT degree. Based on multivariate logistic analysis, gynecologic and allergic emergencies were the only factors significantly correlating with PTSD.

## Authors' contribution

Elham Navab, Maryam Esamaeili and Afshin Khazaei designed the study. Abbas Mogimbeigi analyzed the data and aided in interpreting the results. Finally, all authors discussed the results and contributed to the final manuscript.

Afshin Khazaei1: 0000-0002-8063-3419

Maryam Esmaeili2: 0000-0002-4798-2270

Elham Navab3: 0000-0002-5210-9070
